# Supplementary material for: COZOID: contact zone identifier for visual analysis of protein-protein interactions
Source: BMC Bioinformatics. 2018 Apr 6;19:125. doi: 10.1186/s12859-018-2113-6 (PMC5889581; doi:10.1186/s12859-018-2113-6)
Supplement: Supplementary file 2 — Software build. Executable binary file of the software tool. (ZIP 118,784 kb) [file 12859_2018_2113_MOESM2_ESM.zip › cozoid/java/docs/beansbinding-1.2.1-doc.zip!/org/jdesktop/el/class-use/PropertyNotFoundException.html]

Uses of Class org.jdesktop.el.PropertyNotFoundException (Beans Binding)


---


|  |  |  |
| --- | --- | --- |
| **Overview** \| **Package** \| **Class** \| **Use** \| **Tree** \| **Deprecated** \| **Index** \| **Help** | |  |
| PREV   NEXT | **FRAMES**    **NO FRAMES**     **All Classes** |  |


---


## **Uses of Class org.jdesktop.el.PropertyNotFoundException**

| Packages that use PropertyNotFoundException | |
| --- | --- |
| **org.jdesktop.el** | Customized version of EL for Beans Binding; not for general use. |
| **org.jdesktop.el.impl** | Customized version of EL for Beans Binding; not for general use. |
| **org.jdesktop.el.impl.util** | Customized version of EL for Beans Binding; not for general use. |

Uses of PropertyNotFoundException in org.jdesktop.el

| Methods in org.jdesktop.el that throw PropertyNotFoundException | |
| --- | --- |
| `Expression.Result` | `ValueExpression.getResult(ELContext context, boolean trackResolvedObjects)` |

Uses of PropertyNotFoundException in org.jdesktop.el.impl

| Methods in org.jdesktop.el.impl that throw PropertyNotFoundException | |
| --- | --- |
| `MethodInfo` | `MethodExpressionImpl.getMethodInfo(ELContext context)`             Evaluates the expression relative to the provided context, and returns information about the actual referenced method. |
| `Expression.Result` | `ValueExpressionImpl.getResult(ELContext context, boolean trackResolvedObjects)` |
| `java.lang.Class` | `ValueExpressionImpl.getType(ELContext context)` |
| `java.lang.Object` | `ValueExpressionImpl.getValue(ELContext context)` |
| `java.lang.Object` | `MethodExpressionImpl.invoke(ELContext context, java.lang.Object[] params)`             Evaluates the expression relative to the provided context, invokes the method that was found using the supplied parameters, and returns the result of the method invocation. |
| `boolean` | `ValueExpressionImpl.isReadOnly(ELContext context)` |
| `void` | `ValueExpressionImpl.setValue(ELContext context, java.lang.Object value)` |

Uses of PropertyNotFoundException in org.jdesktop.el.impl.util

| Methods in org.jdesktop.el.impl.util that throw PropertyNotFoundException | |
| --- | --- |
| `static java.beans.PropertyDescriptor` | `ReflectionUtil.getPropertyDescriptor(java.lang.Object base, java.lang.Object property)` |

---


|  |  |  |
| --- | --- | --- |
| **Overview** \| **Package** \| **Class** \| **Use** \| **Tree** \| **Deprecated** \| **Index** \| **Help** | |  |
| PREV   NEXT | **FRAMES**    **NO FRAMES**     **All Classes** |  |


---
